# Type-II Myocardial Infarction – Patient Characteristics, Management and Outcomes

**DOI:** 10.1371/journal.pone.0084285

**Published:** 2014-01-02

**Authors:** Gideon Y. Stein, Gabriel Herscovici, Roman Korenfeld, Shlomi Matetzky, Shmuel Gottlieb, Danny Alon, Natalie Gevrielov-Yusim, Zaza Iakobishvili, Shmuel Fuchs

**Affiliations:** 1 Internal Medicine “B”, Beilinson Hospital, Rabin Medical Center, affiliated to the Sackler School of Medicine, Tel Aviv University, Tel-Aviv, Israel; 2 Cardiology Institute, Chaim Sheba Medical Center, Tel-Hashomer, affiliated to the Sackler School of Medicine, Tel Aviv University, Tel-Aviv, Israel; 3 Neufeld Heart Research Institute, Chaim Sheba Medical Center, Tel-Hashomer, affiliated to the Sackler School of Medicine, Tel Aviv University, Tel-Aviv, Israel; 4 Cardioogy Department, Bikur Cholim Hospital, Jerusalem, Israel; 5 Cardiology Department, Beilinson Hospital, Rabin Medical Center, affiliated to the Sackler School of Medicine, Tel Aviv University, Tel-Aviv, Israel; University of Louisville, United States of America

## Abstract

**Background:**

Type-II MI is defined as myocardial infarction (MI) secondary to ischemia due to either increased oxygen demand or decreased supply. This categorization has been used for the last five years, yet, little is known about patient characteristics and clinical outcomes. In the current work we assessed the epidemiology, causes, management and outcomes of type II MI patients.

**Methods:**

A comparative analysis was performed between patients with type-I and type-II MI who participated in two prospective national Acute Coronary Syndrome Israeli Surveys (ACSIS) performed in 2008 and 2010.

**Results:**

The surveys included 2818 patients with acute MI of whom 127 (4.5%) had type-II MI. The main causes of type-II MI were anemia (31%), sepsis (24%), and arrhythmia (17%). Patients with type-II MI tended to be older (75.6±12 vs. 63.8±13, p<0.0001), female majority (43.3% vs. 22.3%, p<0.0001), had more frequently impaired functional level (45.7% vs. 17%, p<0.0001) and a higher GRACE risk score (150±32 vs. 110±35, p<0.0001). Patients with type-II MI were significantly less often referred for coronary interventions (36% vs. 89%, p<0.0001) and less frequently prescribed guideline-directed medical therapy. Mortality rates were substantially higher among patients with type-II MI both at thirty-day (13.6% vs. 4.9%, p<0.0001) and at one-year (23.9% vs. 8.6%, p<0.0001) follow-ups.

**Conclusions:**

Patients with type-II compared to type-I MI have distinct demographics, increased prevalence of multiple comorbidities, a high-risk cardiovascular profile and an overall worse outcome. The complex medical condition of this cohort imposes a great therapeutic challenge and specific guidelines with recommended medical treatment and invasive strategies are warranted.

## Introduction

In 2007, a joint Task Force of the American College of Cardiology, American Heart Association, European Society of Cardiology and the World Heart Federation published a redefinition of myocardial infarction (MI). [1] Type-II MI was defined as MI secondary to ischemia due to either increased oxygen demand or decreased supply caused by conditions as coronary artery spasm, coronary embolism, anemia, arrhythmias, hypertension, or hypotension. [1] In many of these clinical situations, including sepsis and post-operative state, cardiac troponin is frequently elevated. [2]–[5] The underlying mechanism for this troponin elevation is multifactorial and often indicates myocardial necrosis rather than myocardial ischemia. [3] The incidence of type-II MI among all patients is currently unknown and a rate of 4% was reported among patients who experienced recurrent MI [6]–[8]. However, patient characteristics, clinical presentation, underlying contributing factors, management and outcomes, have not been elucidated.

The Acute Coronary Syndrome Israeli Survey (ACSIS) is a prospective nation-wide consecutive collection of data of acute coronary syndrome patients in Israel. The survey is conducted biennially over a 2-month period and data on all acute coronary syndrome patients in 26 public hospitals in Israel are provided by each participating center by means of the pre-specified case report forms. The Israel Heart Society is responsible for the collection of all case report forms and for maintaining the survey database. [9] Since 2008, the survey has implemented the universal definition of MI. Accordingly, we have performed a comparative analysis between patients with type-I and type-II MI who were enrolled in two consecutive national ACSIS.

## Patients and Methods

### Patient Population

During the 2-month period in 2008 and 2010, detailed data was collected in all 26 ICCU and cardiology wards in all public hospitals in Israel, on patients admitted with the diagnosis of ACS. In addition, data from a representative sample of 37 Internal Medicine wards was collected by the Israel Society of Internal Medicine.

The study population consisted of 2,818 patients with myocardial infraction, of which 2,691 experienced type-I and 127 experienced type-II MI, who were included in the ACSIS registry in 2008 and 2010. Complications of coronary angiography and intervention were documented only in ACSIS 2010.

In-hospital and 30-day outcomes were available for all patients. Mortality at one-year follow-up was available for 93% of the patients. Demographic, historical and clinical data, admission ECG parameters, presence of Q-waves at discharge, medical therapies in-hospital and at discharge, invasive procedures, in-hospital complications and follow-up data were recorded on predefined forms by dedicated physicians. Patients’ functional level was categorized as: normal, mildly impaired or significantly impaired. The existence of anemia was defined at the discretion of the treating physician, based on normal laboratory range in each participating medical center.

### Diagnosis and Definitions of Myocardial Infarction

The diagnosis of type-I and type-II MI were at the discretion of the treating physician, according to the 2^nd^ universal definition of MI. [1] To assure compliance with this definition a retrospective validation of the diagnosis of all type-II MI was performed, independently, by two expert physicians. [1] Patients for whom a specific valid cause for the type-II MI was not established were re-classified as type-I MI. Global Registry of Acute Coronary Events (GRACE) risk score was calculated for each admitted patient [10], [11].

### Ethics Statement

This register-based analysis of pre-existing data was conducted according to the principles expressed in the Declaration of Helsinki. The ACSIS was approved by all the ethical committees in each of the participating medical centers (File S1). Informed consent was specifically waived by the ethical committees of all participating medical centers.

### Statistical Analysis

Statistical analysis was performed using SAS statistical software (version 8.2, SAS Institute, Cary, NC). Categorical variables were expressed as percentage, and continuous variables were expressed as mean ± SD. Comparisons of variables were performed by Chi-Square and Fisher’s exact test for categorical variables and by unpaired t-test test for continuous variables. All tests were two-sided and p value<0.05 was considered statistically significant.

## Results

### Patient Characteristics and Clinical Presentation

Type-II MI was diagnosed in 178 of 2818 patients, of whom, 51 were re-classified as type-I MI because a specific valid cause for the type-II MI was not established. The final cohort of type II MI comprised 127 (4.5%) patients. Compared with type-I, patients with type-II MI were older by an average of 11.5 years and the percentage of females was 2-fold higher (Table 1). Patients with type-II MI more often have a history of coronary revascularization, cardiovascular related comorbidities, and substantially higher rates of chronic renal failure and reduced functionality level (Table 1). GRACE risk score was substantially higher among type II MI patients (150±32 vs. 110±35, p<0.0001), reflecting higher scores both among patients with STEMI (133±34 vs. 96±31, p<0.0001) and NSTEMI (154±30 vs. 123±35, p<0.0001). Clinical presentation varied between the two patient cohorts and patients with type-II were presented more often with atypical symptoms including dyspnea and arrhythmia, diagnosed more often with non-ST elevation MI and were more frequently admitted to an internal medicine ward and less to a cardiology department (Table 2).

**Table 1:** Patient characteristics.

Patient Characteristics	Type-I	Type-II	p
	(n = 2691)	(n = 127)	
Age	63.8±13	75±12	<0.0001
Female (%)	22.3	43.4	<0.0001
BMI	27.6±4.7	25.8±4	0.0009
Current smoker (%)	40.7	15.8	<0.0001
Functionality Level (%)			
Normal	83	54.3	<0.0001
Mildly impaired	12.3	30	
Significantly impaired	4.7	15.7	
GRACE Score	110±35	150±32	<0.0001
Comorbidities (%)			
Prior MI	28.1	44.4	0.0001
Prior Angina Pectoris	32	39.7	NS
Prior PCI	28.1	37.1	0.03
Prior CABG	8.3	14.2	0.02
Heart failure	9.2	25.6	<0.0001
Peripheral vascular disease	8.5	17.3	0.0007
Dyslipidemia	71.5	73.2	NS
Diabetes	35.1	48	0.003
Hypertension	60.6	84.9	<0.0001
Chronic renal failure	12.2	35.7	<0.0001
Past CVA/TIA	8	17.3	0.0002
COPD	7.2	14.8	0.01

BMI – body mass index.

PCI – per-cutaneous intervension.

CABG – coronary artery bypass grafting.

CVA – cerebrovascular event.

TIA – transient ischemic attack.

COPD - chronic obstructive pulmonary disease.

**Table 2:** Presentation of myocardial infarction.

Characteristic	Type-I	Type-II	p
	(n = 2691)	(n = 127)	
First arrival to (%)			
Emergency room	82.4	87.4	<0.0001
Cardiology department*	17.6	12.6	
Hospitalization (%)			
Cardiology	86.2	61.7	<0.0001
Internal Medicine	13.8	38.3	
Presenting symptom (%)			
Typical angina	84.5	54.3	<0.0001
Atypical chest pain	7.5	20.5	<0.0001
Syncope	4.1	5.5	NS
Arrhythmia	4.7	14.2	<0.0001
Dyspnea	3.9	11.8	<0.0001
MI type at presentation (%)			
ST elevation	52.5	19.7	<0.0001
Non ST elevation	44.2	70.1	
Undetermined	3.3	10.2	
MI location (%)			
Anterior	34.2	19.7	<0.0001
Inferior	34.7	25.2	
Lateral	6.9	12.6	
Posterior	1	0.8	
Right ventricle	0.1	0	
Undetermined	23.2	41.7	
Vital signs (mean±SD)			
Systolic blood pressure	141±29	143±33	NS
Diastolic blood pressure	82±17	79±19	NS
Heart rate	80±20	95±25	<0.0001
KILLIP class on admission (%)			
I	85	62.2	<0.0001
II	8.2	24.4	
III	5.1	11	
IV	1.6	2.4	
LV Ejection fraction (%)			
>40%	74.3	65.9	NS
<40%	25.7	34.1	
Blood tests (mean±SD)			
Creatinin (mg/dL)	1.3±0.9	1.6±1.2	0.0002
Hemoglobin (g/dL)	13.8±1.8	11.4±2.2	<0.0001

Intensive or intermediate cardiac care units.

### Causes of Type II MI

Table 3 specifies the main causes for type-II MI. Twenty six percent of the patients had more than one cause (Table 3). The main causes were anemia, followed by sepsis, arrhythmia and post-operation. Sepsis as a cause of type-II MI was more common among patients presenting with STEMI compared with those presenting with NSTEMI (40.7% vs. 19.2%, p = 0.02). Other causes did not differ between STEMI and NSTEMI patients.

**Table 3:** Cause of type-II MI.

Cause	% patients (N = 127)
Anemia	31
Sepsis	24
Arrhythmia	17
Post-operative	14
Hypoxia	14
Heart failure	11
Valvular*	10
Stress**	3
Drugs¶	2
Others§	4
Two causes	18
Three causes	6
Four causes	2

Decompensated aortic stenosis.

Takatsubo, intense pain and suffocation.

Methylphenidate and tadalafil (Cialis).

Vasospasm, extreme hypertension and thyrotoxicosis.

### In-hospital Management

Utilization of revascularization varied between groups (Table 4). Type-II MI patients were less often referred for primary and non-primary angiography and of those who underwent coronary angiography, 50% had undergone PCI. In both groups, the utilization of an invasive strategy showed an inverse relationship to patient’s risk, as assessed by GRACE risk score (Table 5).

**Table 4:** Reperfusion therapy.

	Type-I	Type-II	P
	(n = 2691)	(n = 127)	
Primary reperfusion (%)	69.2	32	<0.0001
PCI	93	87.5	NS
Thrombolysis	6.4	12.5	NS
CABG	0.6	0	NS
Angiography by MI type (%)			
All	88.7	36.2	<0.0001
STEMI	94.3	68	<0.0001
NSTEMI	82.5	28.4	<0.0001
PCI (%)*			
ALL	81.7	50	<0.0001
STEMI	89.5	52.9	<0.0001
NSTEMI	71.8	48.2	<0.0001
Culprit Vessel (%)			
LMCA	1.2	0	NS
LAD	40.3	35.3	
LCX	22.8	26.5	
RCA	34	35.3	
Graft	2.3	2.9	
Complications of PCI (%)			
Closure of side branch	2.8	6.3	NS
Dissection	3.9	6.3	NS
Perforation	0.3	0	NS
No reflow	3.3	6.3	NS
CPR during procedure	1.1	12.5	<0.0001
Urgent CABG	0.5	6.3	0.002

PCI - per cutaneous intervention.

LMCA - left main coronary artery.

LAD - left anterior descending coronary artery.

LCX - left circumflex coronary artery.

RCA - right coronary artery.

CABG - coronary artery bypass graft.

Percentage of all patients who had undergone angiography.

**Table 5:** Differences in GRACE score by MI type and PCI status.

	Type-I		Type-II		p (Type-I vs. Type-II)
	N	Mean±SD	N	Mean±SD	
No-PCI	661	138±35	89	157±28	<0.0001
PCI	1699	99±29	17	116±27	0.016
p (PCI vs. no-PCI)		<0.0001		<0.0001	

PCI - percutaneous coronary intervention.

Patients with type-II MI had more complications during PCI. Specifically, they had undergone more cardio-pulmonary resuscitations and were referred more frequently for urgent CABG. Interestingly, there were no differences in the culprit vessel between MI types (Table 4).

### In and Out of Hospital Outcomes

**Figure 1:**
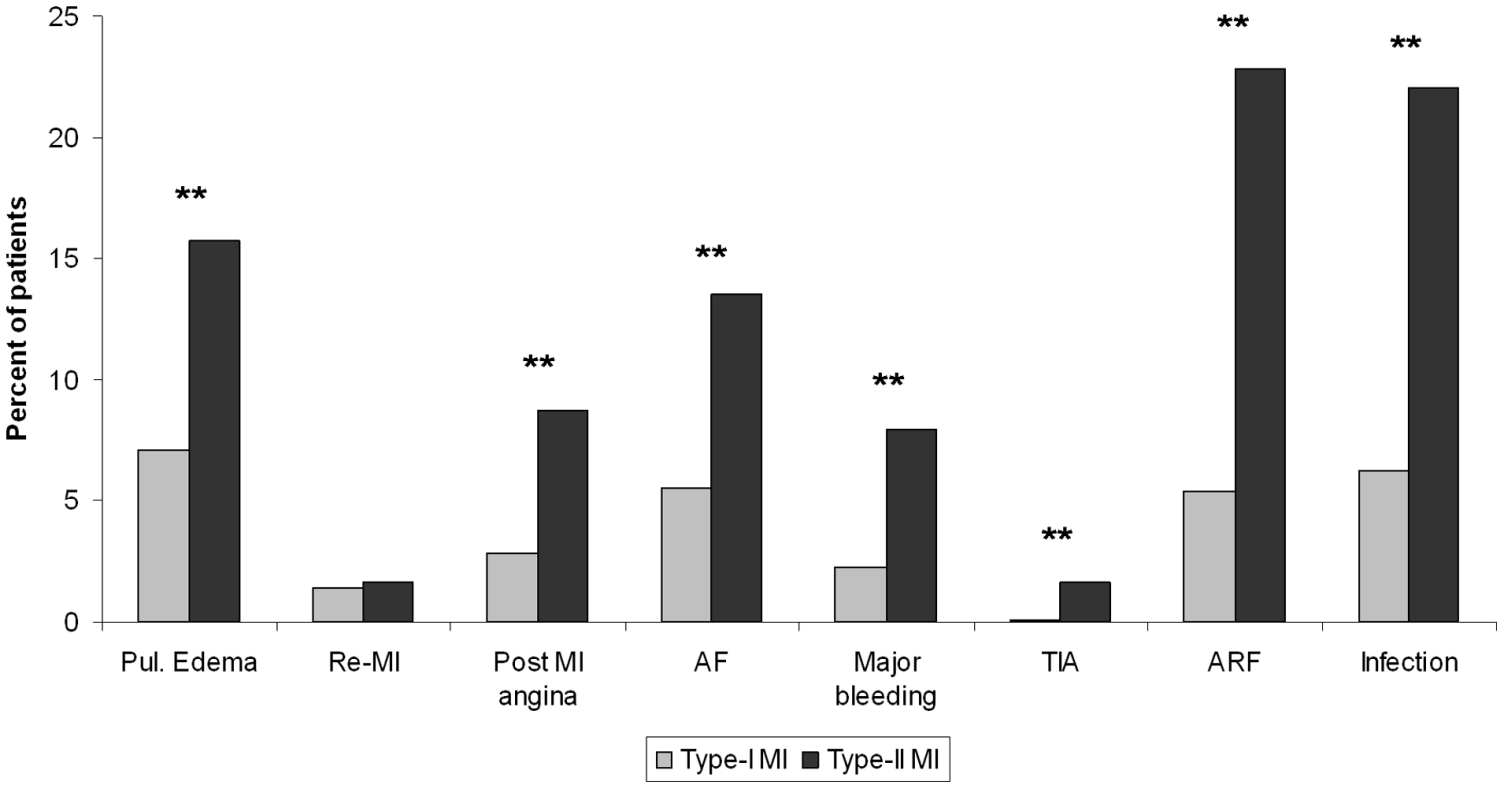
In-hospital complications. In-hospital complications of patients with type-I compared to patients with type-II MI. (** denotes significant difference with p<0.001). Pul. edema - pulmonary edema Re-MI - recurrent myocardial infarction AF - atrial fibrillation TIA - transient ischemic attack ARF - acute renal failure.

**Figure 2:**
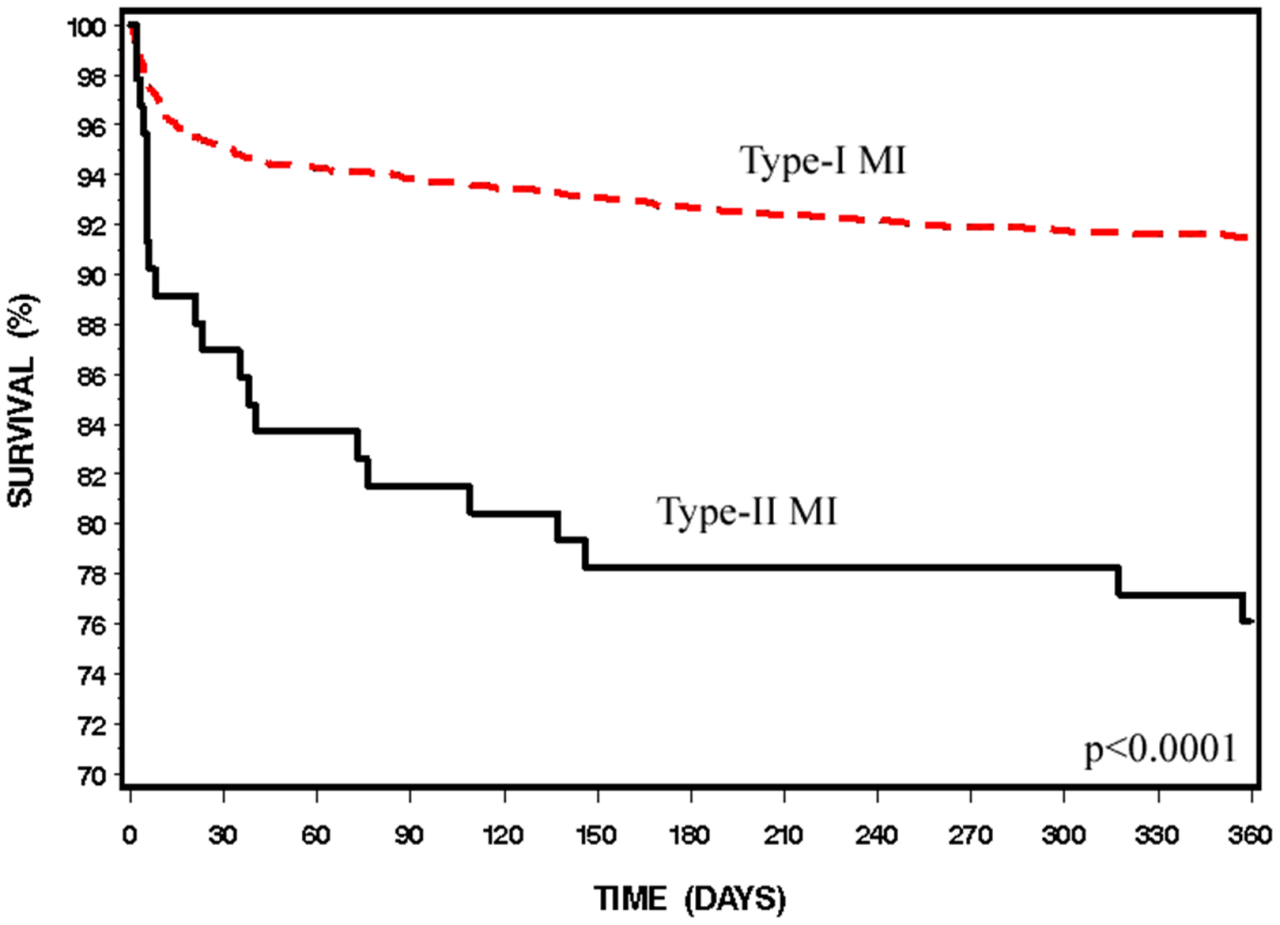
Kaplan-Meier Survival Analysis, Type-I vs. Type-II Myocardial Infarction. Kaplan-Meier survival analysis shows significant differences between groups with overall reduced one-year survival rates among patients with type-II MI (76.1% vs. 91.4%, p<0.0001).

Patients with type-II MI had higher rates of in-hospital complications including post-MI angina and heart failure (Figure 1) and extended hospitalizations (7.5±6.3 vs. 6±5.3 days, p = 0.002). In-hospital and 30-day mortality rates were almost three times higher among patients with type-II compared to type-I MI (11.8% vs. 4.2%, p = 0.0005 and 13.6% vs. 4.9%, p = 0.0005, respectively). Thirty-day major adverse cardiac event rates defined as a composite of death, re-MI, CVA or urgent revascularization, were also significantly higher among patients with type-II MI (18.9% vs. 8.8%, p = 0.0001). Kaplan-Meier survival analysis shows significant differences between groups with overall reduced one-year survival rates among patients with type-II MI (76.1% vs. 91.4%, p<0.0001) (Figure 2). Out-of-hospital to one-year mortality rates were also higher among patients who experienced type-II compared to type I MI (4.4% vs. 12.2%. p<0.0001). Interestingly, patients with type-II MI who had two or more identifiable causes of their MI, compared to those with a single cause, had substantially higher 30-day mortality (30.4% vs. 9.8%, p = 0.009).

Patients with type-II MI less often received guideline-directed medical therapy. These differences were mostly distinctive for clopidogrel but were also significant for four other groups of medications, such as: aspirin, beta-blockers, angiotensin converting enzyme inhibitors/angiotensin receptor blockers and HMG-CoA reductase inhibitors (Table 6).

**Table 6:** Guideline-directed medications at discharge.

	Type-I	Type-II	p
	(n = 2691)	(n = 127)	
Aspirin	97	86	<0.0001
Clopidogrel	86	50	<0.0001
Beta blockers	83	72	0.001
ACE-I/ARB	79	69	0.006
HMG-CoA reductase inhibitors	94	87	0.003

ACE-I - angiotensin converting enzyme inhibitors.

ARB - angiotensin receptor blocker.

## Discussion

The current national prospective survey analysis of close to 3000 patients with MI, demonstrates a near three-fold increase in short- and intermediate-term mortality among patients with type-II compared with type-I MI. This study, the first to characterize patients with type-II MI, shows that these patients, compared to type-I MI, are: 1) older and more frequently female, 2) have higher rates of multiple cardiac and non-cardiac comorbidities with a significantly higher GRACE risk score, 3) more frequently presented with atypical symptoms and diagnosed with NSTEMI, and 4) less frequently referred to coronary interventions and received fewer guideline-directed medications.

In our cohort, type-II MI was diagnosed in 4.5% of all AMI patients and constituted 7% among patients admitted with NSTEMI. These data are in concordance with previous studies assessing the frequency of type II MI in selected populations with previous MI. [6], [7], [12], [13] Nevertheless, following implementation of the 3^rd^ universal definition of MI along with utilization of high-sensitive troponin assays, it is quite possible the population of patients with type-II MI and specially type II NSTEMI will increase [14], [15].

We observed substantial differences in baseline characteristics between patients admitted with type-II compared to type-I MI. Notably, patients with type-II MI are considerably older, more often women, they more frequently have a history of coronary revascularization, chronic renal failure, diabetes and lower functional status. Risk factors for type-II MI in specific clinical situations such as post-operative have been reported and comprised several variables including increased age, dependent functional status and renal failure. [16] It is conceivable, that elderly patients with multiple comorbidities and an underlying coronary disease would be more susceptible to clinical changes that may interfere with the delicate balance of myocardial supply and demand, ensuing in type-II MI.

Therapeutic strategy in the current study was at the discretion of the local medical team. Anemia and sepsis were identified as causes for MI in over 50% of the patients. These clinical conditions, along with frequent presence of chronic renal failure and significantly impaired functional capacity on one hand and the high cardiovascular risk score on the other imposed a great therapeutic decision-making challenge. Hence, additional data are needed in order to draw specific recommendations tailored to the various clinical conditions associated with type II MI. Considering the high cardiovascular risk score of patients with type II MI, recognition of subsets of cohorts, such as those experiencing post-operative MI, may allow to implement invasive strategy [2], [11], [17]–[19]. On the other hand, many of these patients may require initial stabilization of the cause of the MI. This obligatory time lag may dictate conservative approach with utilization of delayed invasive strategy only in selective patients. [20] Whether, in selected patient populations, a more invasive approach would benefit patients with type II MI has not been studied. Similarly, whether current recommended medical treatments would advantage patients with type II MI is at present unknown. Considering the complexity of this cohort, a potential important implication of our study is the need for dedicated studies to assess comprehensive therapeutic strategies in this growing patient population.

Short-term and intermediate outcomes differ between patients with type-II and type-I MI. In-hospital complications were substantially more frequent and short-term and intermediate mortality rates were near three-fold higher, reaching 13.6% and 23.9% at 30 days and 1-year, respectively. Similar 30-day mortality rates were previously noted in patients with perioperative MI. [2] Thus, our data further extends the relatively high mortality rates to a broader type-II patient population.

### Limitations

The current study carries several limitations. In this national survey, patients admitted to non-cardiac intensive care units were not included. Thus, both the true frequency of type-II MI and the observed mortality rates, which are usually substantial among medical intensive-care patients, may be higher. Invasive strategy was at the discretion of the treating physicians and overall, only a minority of type-II MI patients were referred to coronary angiography. Specific reasons for such decisions were not gathered and accordingly, no recommendation regarding patient selection can be made. The relatively small number of type-II MI patients limits the power of our study and preclude multivariate analysis to identify predictors for and risk-stratification of type-II MI. Importantly, re-classification of patients with type II to type I MI was performed in 28% of our cohort. Precise distinction between type II and type I MI in daily practice may be perplexing as many of the causes of type II MI may actually be a complication of type I MI (i.e. arrhythmia, heart failure) and other, such as surgery or inflammatory state may, by themselves, lead to plaque rupture.

### Conclusions

Type-II MI is not infrequent, especially among patients with NSTEMI. Compared to type-I MI, it is more frequent among the elderly with multiple comorbidities, high ACS risk score and associated with increased short and intermediate-term mortality. The implementation of current ACS guidelines to patients with type-II MI is challenging and more evidence-based patient-tailored therapeutic strategies are warranted.

## Supporting Information

File S1 Participating medical centers in the ACSIS registry.
(DOCX)
