# Supplementary material for: Type-II Myocardial Infarction – Patient Characteristics, Management and Outcomes
Source: PLoS One. 2014 Jan 2;9(1):e84285. doi: 10.1371/journal.pone.0084285 (PMC3879301; doi:10.1371/journal.pone.0084285)
Supplement: File S1 — Participating medical centers in the ACSIS registry. (DOCX) [file pone.0084285.s001.docx]

### Supporting Methods

### S1 – Participating medical centers in the ACSIS registry

Afula - Central Hae’mek; Ashkelon - Barzilai; Be’er Ya’aqov - Assaf Harofeh; Be’er Sheva - Soroka; Eilat - Josephtal; Hadera - Hillel Yaffe; Haifa - B’nei-Zion, Rambam, Carmel; Holon - Wolfson; Jerusalem - Bikur Holim, Sha’arei Zedek, Hadassah Mount Scopus, Hadassah Ein Kerem; Kfar Saba - Meir; Nahariyah - Western Galilee; Nazareth - EMMS Hospital, Holy Family; Netanya - Laniado; Petah Tikva - Rabin Beilinson, Rabin Golda, Ramat Gan - Sheba; Rehovot - Kaplan; Tel Aviv - Sourasky; Tiberias - Poriah; Zefat - Rebecca Sieff.
